# Consecutive melon monocropping is associated with microbial community homogenization and reduced bacterial network complexity

**DOI:** 10.3389/fmicb.2026.1884506

**Published:** 2026-07-27

**Authors:** Guozhi Hu, Muxi Xie, Guangzhou Wang, Jiangzhou Zhang, Junling Zhang

**Affiliations:** 1 Institute of Fruits and Vegetables, Xinjiang Academy of Agricultural Sciences, Urumqi, China; 2 State Key Laboratory of Nutrient Use and Management, Key Laboratory of Plant-Soil Interactions, Ministry of Education, College of Resources and Environmental Sciences, National Academy of Agriculture Green Development, China Agricultural University, Beijing, China; 3 Inner Mongolia Key Laboratory of Soil Quality and Nutrient Resources, Key Laboratory of Agricultural Ecological Security and Green Development at Universities of Inner Mongolia Autonomous, College of Resources and Environmental Sciences, Inner Mongolia Agricultural University, Hohhot, China

**Keywords:** consecutive monocropping, ecosystem function, fungal pathogen, microbial community, soil health

## Abstract

Soil microbiomes are critical for sustaining ecosystem functioning and soil health. Monocropping often disrupts soil microbial communities, leading to yield penalties and soil degradation. Although bio-organic fertilizers are widely used to alleviate the negative effects of monocropping, their long-term associations with microbial community structure and ecosystem functioning across monocropping durations remain insufficiently understood. Here, we collected 72 soil samples from Hami melon (*Cucumis melo*) fields at three sites in Turpan, northwestern China. All sampled fields received *Bacillus subtilis*–based bio-organic fertilizer and consisted of independently managed 1-year (M1), 3-year (M3), and 5-year (M5) monocropping systems sampled within the same year. Across fields with longer monocropping durations, key putative fungal pathogens, including *Fusarium*, *Monosporascus*, and *Alternaria*, showed lower relative abundances, whereas potentially beneficial bacterial taxa such as *Bacillus*, *Paenibacillus*, *Sphingomonas*, and *Lysobacter* were enriched. However, both bacterial and fungal communities exhibited reduced Bray–Curtis dissimilarity and β-dispersion, consistent with increasing microbial homogenization. Although the overall phylogenetic diversity of bacterial and fungal communities remained comparatively stable, the relative abundance and richness of rare taxa declined across monocropping durations. In addition, bacterial co-occurrence networks exhibited reduced complexity in fields with longer monocropping durations, while fungal subnetworks remained comparatively stable. Among the evaluated soil ecosystem functions, carbon degradation exhibited a non-linear response pattern and reached its highest value in M3, whereas nutrient cycling, carbon storage, and physical structure remained relatively stable across monocropping durations. Overall, our findings suggest that prolonged monocropping combined with long-term *Bacillus subtilis*–based bio-organic fertilization is associated with microbial community homogenization and simplified bacterial network structures, while most soil ecosystem functions remain comparatively stable.

## 1 Introduction

Soil health is closely linked to the soil’s capacity to sustain primary productivity in natural and cropland ecosystems (Lehmann et al., 2020; Qiao et al., 2022; Romero et al., 2024). As a typical cultivation in modern agriculture, consecutive monocropping often leads to accumulation of pests and pathogens, reduction of beneficial microorganisms (Li et al., 2014; Wang et al., 2019), inhibition of carbon and nitrogen mineralization (She et al., 2017; Wyngaard et al., 2012) and ultimately weakening soil health (Batista et al., 2024; Mulumba et al., 2012). Melon is an important global cash crop, and China is one of the main melon-producing and -consuming countries (Li et al., 2023). However, consecutive monocropping of melon has led to soil degradation and yield penalties (Liao and Xia, 2024). Major soil-borne putative fungal pathogens such as *Fusarium*, *Alternaria*, and *Monosporascus* are reported to threaten Hami melon production in the consecutive monocropping (Aleandri et al., 2017; Hanson et al., 2018; Naeem et al., 2019). The application of bio-organic fertilizer is shown to be effective in alleviating these adverse effects by regulating microbial communities (Li H. L. et al., 2024; Yang et al., 2025). However, whether continuous application of bio-organic fertilizers can sustainably suppress soil putative fungal pathogens and directly modulate microbial communities in melon monocropping systems remains unclear.

The dynamics of microbial community structure are governed by microbial assembly processes. Both deterministic processes (homogeneous and heterogeneous selection) and stochastic processes (homogenizing dispersal, dispersal limitation and drift) shape microbial community structure (Chave, 2004; Chesson, 2000; Ning et al., 2020). A previous study has shown that agricultural conversion reduces both taxonomic and functional β-diversity, leading to biotic homogenization at continental and global scales (Peng Z. et al., 2024). In the tobacco rhizosphere, bacterial community assembly exhibits a temporal pattern, transitioning from stochastic dominance in the first year of monocropping to deterministic processes, and returning to stochastic dominance after 10 years (Zhou F. et al., 2025). Notably, the diversity and relative abundance of abundant, moderate, and rare taxa are affected by changes in microbial composition and structure, with potential consequences for soil ecosystem functions. Arable farming drives fungal community homogenization by increasing the dominance of abundant taxa while disproportionately reducing rare, narrowly distributed fungal groups (Banerjee et al., 2024). Furthermore, monocropping often reduces the complexity of soil microbial networks relative to intercropping systems and short-term monoculture (Li et al., 2025; Peng Y. M. et al., 2024). Therefore, disentangling how microbial assembly, taxa dynamics, and network complexity respond to long-term monocropping is essential for understanding the ecological consequences of agricultural intensification.

Soil ecosystem functions, including primary productivity, carbon sequestration, organic matter decomposition, soil fertility, water conservation, biological habitat provision, and climate regulation, are closely linked to soil health (Creamer et al., 2022; Li Y. et al., 2024; Zhou T. et al., 2025). Alterations in microbial community assembly, taxonomic composition, and network complexity can have profound effects on soil ecosystem functioning. Deterministic assembly processes influence ecosystem functioning through environmental filtering, which favors taxa adapted to specific ecological conditions and may enhance ecosystem performance when functionally important species are selected, whereas functionality may decline when less efficient taxa dominate (Graham et al., 2017; Knelman and Nemergut, 2014). Notably, rare taxa, despite their low abundance, are highly sensitive to environmental change and exert a disproportionate influence on soil biological functions (Jousset et al., 2017; Ziegler et al., 2018). Microbial network complexity also influences soil functions, with more complex microbial networks often being associated with higher ecosystem functioning (Yang et al., 2023; Zhai et al., 2024). Hence, understanding the temporal dynamics of microbial community assembly, changes in different microbial taxa, and network complexity is crucial for alleviating biological constraints in such system.

In the present study, we collected soil samples from independently managed Hami melon (*Cucumis melo*) fields at three sites in Turpan, the main melon-producing region of Xinjiang Province, China, representing 1-, 3-, and 5-year continuous monocropping systems. Soil ecosystem functions, including physical structure, carbon storage, carbon degradation, and nutrient cycling were quantified. We hypothesized that (1) fields with longer monocropping durations under long-term bio-organic fertilization would exhibit lower relative abundances of putative fungal pathogens; (2) microbial communities across fields differing in monocropping duration would show increased homogenization, reduced rare taxa, and simplified bacterial and fungal network structures; and (3) shifts in rare taxa and microbial network structure would be associated with changes in soil ecosystem functions.

## 2 Materials and methods

### 2.1 Study site and soil sample collection

The sampling sites were located in Gaochang District and Shanshan County, Turpan, Xinjiang Province, China. Gaochang district had two experimental regions, Sanbaoxiang town (42°50′56′′–42°55′17′′ N, 89°25′4′′–89°21′32′′ E) and Yaerzhen town (42°50′53′′–42°55′14′′ N, 89°18′21′′–89°21′32′′ E), and Shanshan county has one experimental region, Tuyugou town (42°45′34′′–42°48′58′′ N, 89°33′19′′–89°37′55′′ E). With an average annual temperature of 13.4 °C and annual precipitation of 16.6 mm, the regional climate is distinctly arid. All sampling sites had a history of nearly 10 years of continuous production of table grapes before planting Hami melons at these sites. At these sites, soil samples were collected from independently managed Hami melon fields with 1 (M1), 3 (M3), and 5 (M5) years of consecutive monocropping. Each treatment received the same *Bacillus subtilis*–based bio-organic fertilizer. Specifically, well-composted chicken manure was applied as a basal fertilizer at a rate of 45 m^3^ ha^–1^ before planting by thoroughly mixing it with fine soil and incorporating it into the field through rotary tillage, while a *Bacillus subtilis*–based bio-organic fertilizer was applied as a topdressing at an average rate of 75 kg ha^–1^ during the flowering stage of melon using an integrated water–fertilizer irrigation system. At each site, there were eight replicate plots for each treatment. From each plot, five 20-cm-deep soil cores were gathered and mixed to create a composite sample. All bulk soil samples were partitioned into three sub-samples. The first part (∼50 g) was stored at 4 °C for soil extracellular enzyme activity and soil basal respiration, the second (∼300 g) was air-dried for determination of soil physicochemical properties and the third was stored at −80 °C for DNA extraction.

### 2.2 Soil physicochemical and biological properties measurement

Measured physical soil health indicators included surface hardness, subsurface hardness and bulk density. A penetrometer was used to obtain soil hardness at two depths: surface (0–10 cm) and subsurface (10–20 cm) (Zhang et al., 2023). Soil bulk density was calculated by measuring the mass of wet soil samples, then drying samples at 105 °C for 48 h and measuring mass of the dry soil samples (Al-Shammary et al., 2018). Determination of chemical indicators (pH, electrical conductivity, total carbon, total nitrogen, NH_4_^+^-N, NO_3_^–^-N, available nutrients of P, K, B, Fe, Mn, Cu and Zn, and exchangeable nutrients of Ca, Mg and Na) followed the methods described in a previous study (Zhang et al., 2023).

Soil organic carbon (SOC) was determined by dichromate oxidation titration, and permanganate oxidizable carbon (POXC) was measured according to the permanganate oxidation protocol (Weil et al., 2003). Extractable proteins were quantified spectrophotometrically by the Bradford method in 96-well microplates (Wright and Upadhyaya, 1996). Soil basal respiration (CO_2_ emission) was determined by incubating 10 g of soil in 100 mL sealed vials (24 h at 25 °C) followed by gas chromatograph (Agilent 7890A, Santa Clara, CA, USA). The activities of carbon cycling-related enzymes (α-1,4-glucosidase, β-1,4-glucosidase, β-cellobiosidase and β-1,4-xylosidase), nitrogen cycling-related enzymes (β-1,4-N-acetylglucosaminidase and L-leucine-aminopeptidase), phosphorus cycling-related enzymes (acid phosphatase) and sulfur cycling-related enzymes (sulfatase) were measured using the microplate-scale fluorometric method (Bell et al., 2013; Marx et al., 2001).

### 2.3 Soil DNA extraction, sequencing and bioinformatic analysis

Total DNA was extracted from 0.5 g samples using the MoBio PowerSoil kit (San Mateo, CA, USA) and preserved at −80 °C for later use. The microbial community was analyzed by amplifying the bacterial 16S rRNA gene V4 region using primers 515F (5′-GTGCCAGCMGCCGCGGTAA-3′) and 806R (5′-GGACTACHVGGGTATCTAAT-3′), and the fungal ITS region using primers ITS3F (5′-GCATCGATGAAGAACGCAGC-3′) and ITS4 (5′-TCCTCCGCTTATTGATATGC-3′). PCR amplification was performed under the following thermal cycling conditions. For bacteria, an initial denaturation at 95 °C for 5 min, then 37 cycles of denaturation at 95 °C for 45 s, annealing at 50 °C for 30 s and extension at 72 °C for 45 s, followed by a final extension at 72 °C for 10 min. For fungi, an initial denaturation at 94 °C for 3 min, then 30 cycles of denaturation at 94 °C for 45 s, annealing at 50 °C for 30 s, and extension at 72 °C for 45 s, followed by a final extension at 72 °C for 10 min. All PCR products were purified and sequenced on an Illumina MiSeq platform (Illumina, San Diego, CA, USA).

Raw sequences were quality-filtered using the Quantitative Insights Into Microbial Ecology 2 toolkit (Bolyen et al., 2019). Primer removal, quality filtering, truncation, dereplication, denoising and chimera removal were performed using the DADA2 pipeline. The acquired high-quality sequences were split into amplicon sequence variants (ASVs). The taxonomic assignment was made using the SILVA 16S rRNA database for bacteria (Pruesse et al., 2007) and the UNITE database for fungi (Nilsson et al., 2019). The functional annotation of fungi was performed using the FungalTraits database (Polme et al., 2020). The ASV tables were rarefied to the minimum sequencing depth per sample: 26,920 bacterial high-quality sequences and 40,986 fungal high-quality sequences were obtained. Rarefaction curves were produced using R package “vegan” (Dixon, 2003).

### 2.4 Determination of soil ecosystem functions

We used averaged and single function approaches (Byrnes et al., 2014; Delgado-Baquerizo et al., 2017) for quantifying carbon degradation, carbon storage, nutrient cycling, physical structure. Carbon degradation was quantified based on the activities of carbon-acquiring extracellular enzymes, including α-glucosidase (AG), β-glucosidase (BG), β-cellobiosidase (BC), and β-xylosidase (BX), together with soil basal respiration (Delgado-Baquerizo et al., 2016; Zhou T. et al., 2025). Carbon storage was defined by total carbon (TC), SOC and POXC. Nutrient cycling included total nitrogen (TN), NH_4_^+^, NO_3_^–^, available phosphorus (AP), available potassium (AK), available B, Fe, Mn, Cu and Zn, exchangeable Ca and Mg, as well as protein content and nutrient-acquiring enzyme activities, including β-1,4-N-acetylglucosaminidase (NAG), L-leucine aminopeptidase (LAP), acid phosphatase (ACP), and arylsulfatase (Sul). All above mentioned parameters were considered “more is better.” Soil physical structure referred to soil surface hardness, subsurface hardness and bulk density, which were considered “less is better.” All indicators were standardized to a 0–1 scale following the multifunctionality framework, and equal weights were assigned to all indicators within each functional category. Functional scores were then calculated as the arithmetic mean of the standardized indicator values. Finally, all single ecosystem functions were quantified by computing the average of normalized values (Maestre et al., 2012; Zhou T. et al., 2025).

### 2.5 Statistical analysis

The distance-decay relationships were evaluated by correlating geographic distance with community dissimilarity (Sørensen similarity index) using the “geosphere” package. Faith’s phylogenetic diversity (PD) was calculated from the sequence-based phylogenetic tree, with higher PD typically reflecting increased functional breadth within the community (Faith, 1992; Lozupone and Knight, 2008). The PD of microbial community was calculated after rarefying using the “vegan” package (Dixon, 2003; Xie et al., 2024). Principal coordinate analysis was performed by computing weighted Bray–Curtis distance matrices to explore the difference in bacterial and fungal communities through the “vegan” package (Borcard et al., 2011; Dixon, 2003). Permutational multivariate analysis of variance (PERMANOVA) (Anderson, 2001) was conducted to explore the effects of sampling sites and monocropping duration on soil community composition using the “adonis” function of the “vegan” package (Dixon, 2003). The Kruskal–Wallis test, a non-parametric method implemented in the “stats” package (Gerald, 1991) was used to evaluate the differences in community dissimilarity among different monocropping durations. To evaluate treatment-group β-dispersion, ASV tables were Hellinger-transformed prior to distance calculation. β-dispersion was quantified as the distance of individual samples to their group centroids using the betadisper function in the vegan package (Dixon, 2003). Differences in β-dispersion among treatments were assessed using the Kruskal–Wallis test. To quantify the relative contributions of stochastic versus deterministic processes in microbial community assembly across varying monocropping durations, we used the phylogenetic bin-based null model analysis for both bacterial and fungal communities (Ning et al., 2020). Community assembly processes were assessed using the phylogenetic null model framework based on β-nearest taxon index (βNTI) and Bray–Curtis-based Raup–Crick (RC_*Bray*_) metrics with 1,000 randomizations. βNTI > +1.96 and βNTI < −1.96 indicated heterogeneous selection and homogeneous selection, respectively, whereas | βNTI| < 1.96 combined with RC_*Bray*_ values (>+0.95, <−0.95, or between −0.95 and +0.95) indicated dispersal limitation, homogenizing dispersal, and drift.

Adapted from previous studies (Chen et al., 2019; Dai et al., 2016), rare, moderate, and abundant taxa were classified based on the mean relative abundance of each ASV across all samples, defining abundant taxa as those with >0.1% mean relative abundance, rare taxa as <0.01%, and moderate taxa as 0.01%–0.1%. Linear discriminant analysis effect size (LEfSe) was employed to identify bacterial and fungal biomarkers differentiating the three consecutive monocropping duration treatments (Wu et al., 2021). The co-occurrence network was constructed using the “ggClusterNet” package based on Spearman correlations (Wen et al., 2022). ASVs used for constructing co-occurrence networks with a mean relative abundance < 0.01% across all samples were removed (Jiao et al., 2020). Correlations were calculated after Trimmed Mean of M-values (TMM) normalization, and only significant associations with a Spearman correlation coefficient |r| > 0.8 and *P* < 0.05 were retained for network construction (Yuan et al., 2021; Zhou et al., 2010). A total of 590 bacterial and fungal ASVs were included in the network analysis. Network topological properties, including average degree, degree centrality, betweenness centrality, and total edges, were calculated using the “igraph” package. Topological features were subsequently calculated for each subnetwork using the same approach (Ma et al., 2016).

To analyze the effect of monocropping duration on single ecosystem functions and microbial alpha diversity, we fitted a linear mixed-effects model (LMM) [e.g., carbon degradation ∼ monocropping duration + (1| Site)] using the “lmer” function in the “lme4” package in R (Bonate, 2011; De Boeck et al., 2011). In these models, monocropping duration was included as a fixed effect, while sampling site was treated as a random effect. The significance of the fixed effect (monocropping duration) was tested using the Kenward–Roger approximation for degrees of freedom, which provides more accurate *F*-tests in linear mixed-effects models by adjusting for small-sample bias and complex random-effects structures. Upon identifying a significant main effect, we conducted *post hoc* pairwise comparisons between treatments using the “emmeans” package with Tukey’s HSD adjustment for multiple comparisons (Hou et al., 2021).

## 3 Results

### 3.1 Dynamics of putative fungal pathogens and beneficial bacterial taxa

**FIGURE 1:**
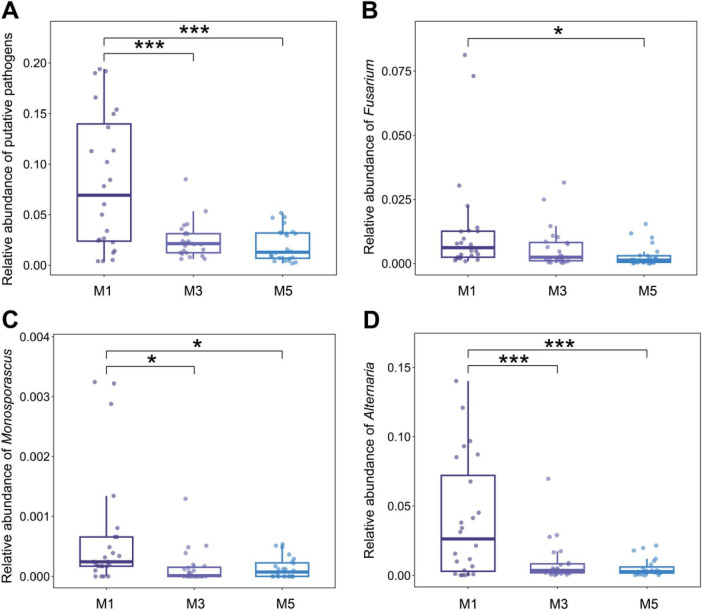
Relative abundances of putative fungal pathogens, *Fusarium*, *Monosporascus* and *Alternaria* at different monocropping durations of M1 (year 1), M3 (year 3) and M5 (year 5). Boxplots and raw data points of total putative fungal pathogens **(A)**, *Fusarium* **(B)**, *Monosporascus* **(C)** and *Alternaria* **(D)** under different monocropping durations. Boxplots **(A–D)** indicate median (box center line), 25th (box bottom line), 75th (box top line) percentiles, and 5th (the lower whisker) and 95th (the upper whisker) percentiles. Significant differences among treatments are determined based on estimated marginal means (±95% confidence intervals) derived from the linear mixed-effects model followed a *post hoc* Tukey’s HSD test. Significance is indicated by *P < 0.05, ***P < 0.001.

Based on the mixed-effects models, the relative abundance of putative fungal pathogens differed significantly among fields with different monocropping durations. The M1 treatment exhibited the highest abundance of total putative fungal pathogens, whereas pathogen abundance was significantly reduced in M3 and M5 (Figure 1A). Similar patterns were observed for the major pathogenic fungal genera *Fusarium*, *Monosporascus* and *Alternaria*. For instance, the relative abundance of the genus *Fusarium* was significantly lower in M5 compared with M1 (*P* < 0.05; Figure 1B), while genus *Monosporascus* showed a pronounced decline from M1 to both M3 and M5 (*P* < 0.05; Figure 1C).

**FIGURE 2:**
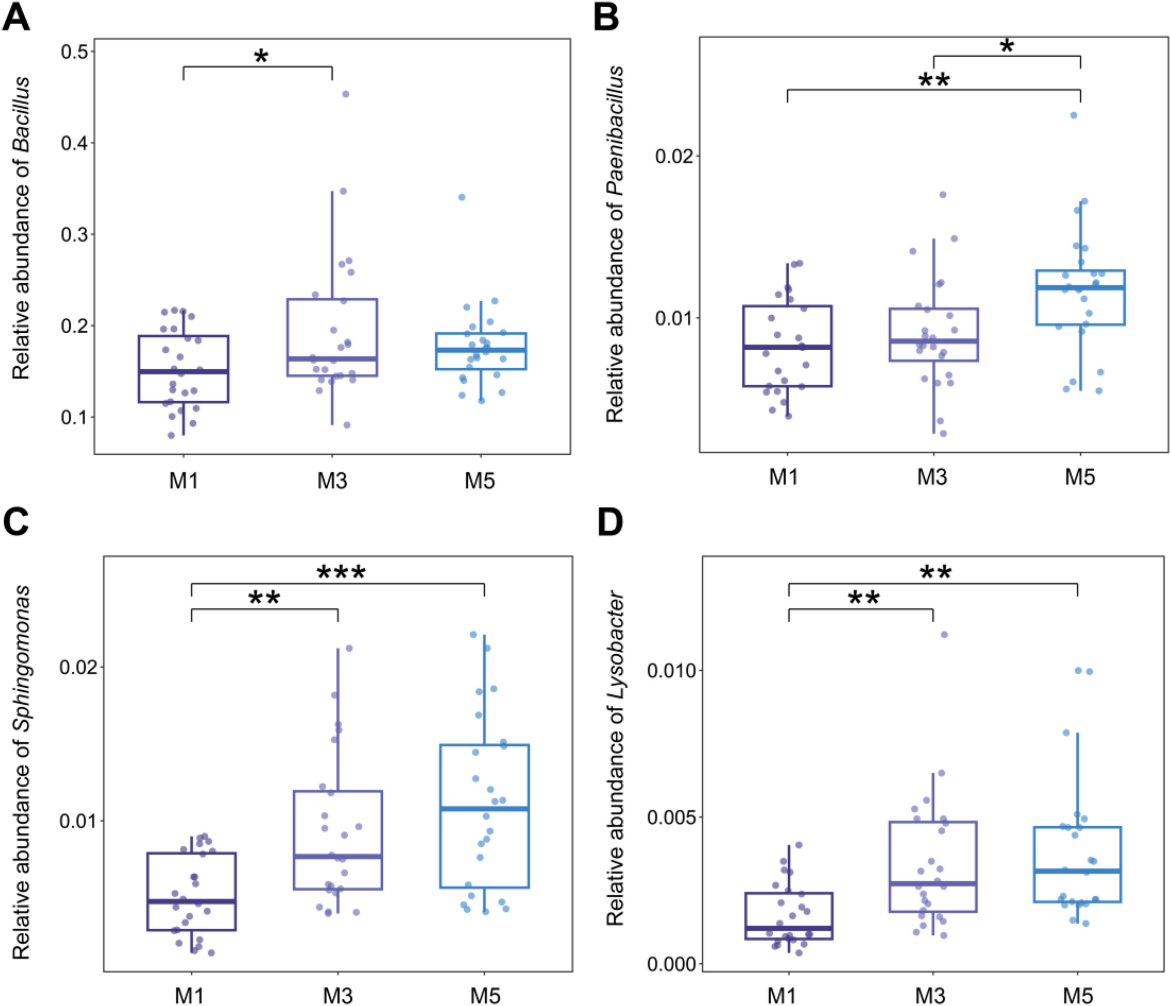
Relative abundances of some beneficial bacterial taxa at different monocropping durations of M1 (year 1), M3 (year 3) and M5 (year 5). Boxplots and raw data points of *Bacillus* **(A)**, *Paenibacillus* **(B)**, *Sphingomonas* **(C)** and *Lysobacter* **(D)** under different monocropping durations. Boxplots **(A–D)** indicate median (box center line), 25th (box bottom line), 75th (box top line) percentiles, and 5th (the lower whisker) and 95th (the upper whisker) percentiles. Significant differences among treatments are determined based on estimated marginal means (±95% confidence intervals) derived from the linear mixed-effects model followed a *post hoc* Tukey’s HSD test. Significance is indicated by *P < 0.05, **P < 0.01, ***P < 0.001.

Several potentially beneficial bacterial taxa also varied significantly across monocropping durations (Figure 2). Bacillus was enriched in M3 relative to M1 (*P* < 0.05), whereas no significant differences were detected between M5 and the other treatments (Figure 2A). In contrast, *Paenibacillus* exhibited a significantly higher relative abundance in M5 than in both M1 and M3 (*P* < 0.05), while no significant difference was observed between M1 and M3 (Figure 2B). Similar trends were observed for *Sphingomonas* and *Lysobacter*, both of which were significantly enriched in M5 compared with M1 (*P* < 0.01), whilst M3 had higher relative abundances than M1 (Figures 2C,D).

Further analysis revealed significant negative correlations between the relative abundances of four potentially beneficial bacterial taxa and total putative fungal pathogens (Supplementary Figure 1). In particular, the relative abundances of *Fusarium* and *Alternaria* were also significantly negatively correlated with those of beneficial bacterial taxa such as *Lysobacter*, *Paenibacillus* and *Sphingomonas*, whereas no significant correlations were detected between *Monosporascus* and other potentially beneficial bacterial taxa (Supplementary Figure 1).

### 3.2 Microbial community dissimilarity and microbial assembly in response to monocropping durations

**FIGURE 3:**
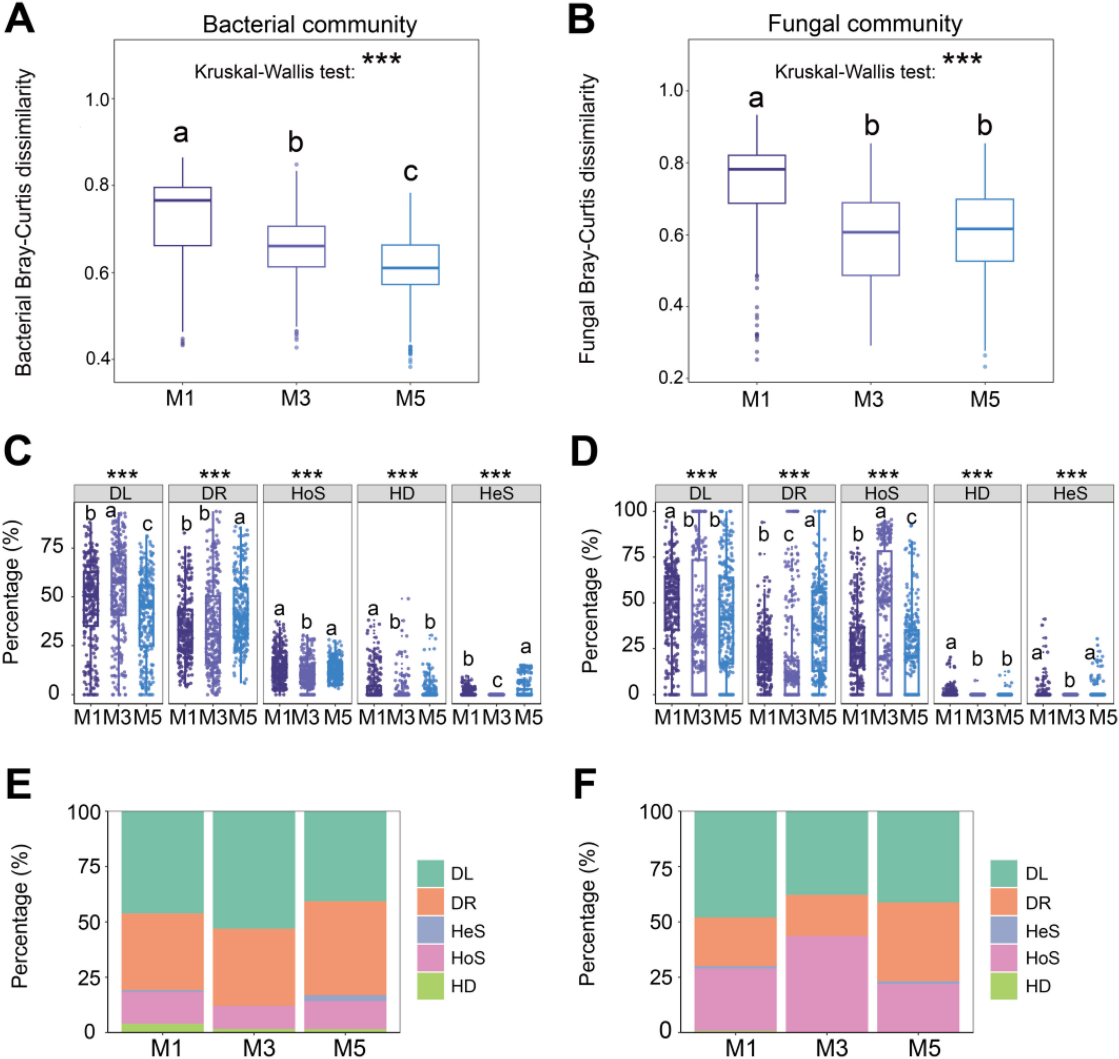
Bray–Curtis dissimilarity of bacterial **(A)** and fungal **(B)** communities at different monocropping durations of M1 (year 1), M3 (year 3) and M5 (year 5), with statistical differences indicated by different letters. Relative contributions of five ecological processes (DL, dispersal limitation; DR, drift; HoS, homogeneous selection; HD, homogenizing dispersal; and HeS, heterogeneous selection) to the assembly of bacterial community **(C)** and fungal community **(D)** under M1, M3 and M5. Statistical differences were based on Kruskal–Wallis tests (***P < 0.001), whereas different lowercase letters indicate significant pairwise differences among monocropping durations based on Wilcoxon rank-sum tests with FDR correction. Percentages of five ecological processes in bacterial **(E)** and fungal community **(F)** at different monocropping durations of M1 (year 1), M3 (year 3) and M5 (year 5).

Distance-decay relationships were observed between microbial community similarity and geographic distance (Supplementary Figures 2A,B). Principal coordinate analysis revealed clear differentiation of both bacterial and fungal communities across monocropping durations (Supplementary Figures 2C,D). Consistently, PERMANOVA indicated that monocropping duration significantly explained variation in both bacterial and fungal community composition (Supplementary Table 1). Importantly, Bray–Curtis dissimilarities of both bacterial and fungal communities gradually decreased across fields with longer monocropping durations (Figures 3A,B). For bacterial communities, β-dispersion differed significantly among monocropping durations (Supplementary Figure 2E). M1 exhibited the highest β-dispersion, indicating greater within-community heterogeneity, whereas M3 and M5 showed significantly lower values, consistent with greater community homogenization in fields with longer monocropping durations. Similarly, fungal communities displayed the highest heterogeneity in M1, while β-dispersion was lower in M3 and M5 (Supplementary Figure 2F). Overall, both bacterial and fungal communities showed reduced within-community heterogeneity across fields with increasing monocropping duration.

To characterize overall assembly patterns, microbial assembly processes were evaluated across all samples from the three study sites. In general, stochastic rather than deterministic processes dominated the assembly of both bacterial and fungal communities (Figures 3C–F). In bacterial communities, dispersal limitation was highest in M3, whereas the relative contribution of ecological drift was greater in M5 than in M1 and M3 (Figure 3C). In contrast, homogeneous and heterogeneous selection contributed least in M3 (Figures 3C,E).

Fungal community assembly exhibited stronger variation among monocropping durations. M1 showed the highest relative contribution of dispersal limitation and homogenizing dispersal, whereas ecological drift was greatest in M5 (Figures 3D,F). In contrast, M3 exhibited the highest contribution of homogeneous selection and the lowest contribution of heterogeneous selection (Figures 3D,F). Stochastic processes dominated bacterial community assembly, with dispersal limitation and drift together accounting for more than 80% across all monocropping durations (Supplementary Table 2). In contrast, fungal community assembly showed a stronger influence of homogeneous selection, particularly in M3 (43.7%), compared with M1 (28.1%) and M5 (21.9%) (Supplementary Table 2).

### 3.3 Dynamics of abundant, moderate, rare taxa and biomarkers in response to different monocropping durations

**FIGURE 4:**
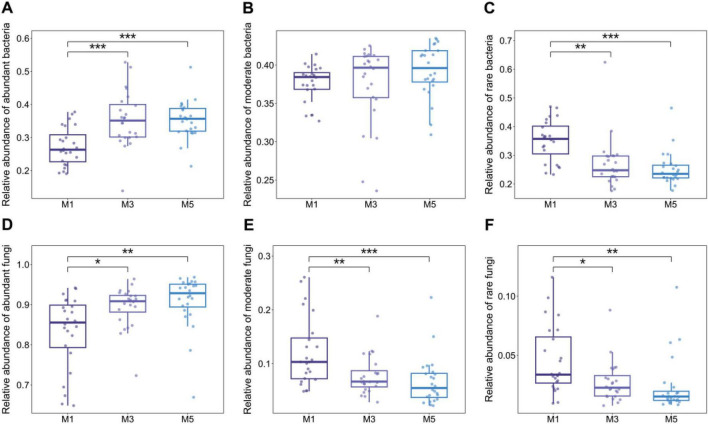
Boxplots and raw data points of abundance of abundant bacteria **(A)**, abundance of moderate bacteria **(B)**, abundance of rare bacteria **(C)**, abundance of abundant fungi **(D)**, abundance of moderate fungi **(E)** and abundance of rare fungi **(F)** at different monocropping durations of M1 (year 1), M3 (year 3) and M5 (year 5). Boxplots indicate median (box center line), 25th (box bottom line), 75th (box top line) percentiles, and 5th (the lower whisker) and 95th (the upper whisker) percentiles. Significant differences among treatments were determined based on estimated marginal means (±95% confidence intervals) derived from the linear mixed-effects model followed a *post hoc* Tukey’s HSD test. Significance is indicated by *P < 0.05, **P < 0.01, ***P < 0.001.

Linear mixed-effects models (LMMs) showed that the phylogenetic diversity of both bacterial and fungal communities did not differ significantly across monocropping durations (Supplementary Figure 3). In contrast, when bacterial and fungal communities were further classified into abundant, moderate, and rare taxa, significant differences in community composition were observed among treatments. The relative abundances of abundant bacterial and fungal taxa were significantly higher in M3 and M5 than in M1, whereas moderate fungal taxa as well as rare bacterial and fungal taxa showed higher relative abundances in M1 than in M3 and M5 (Figure 4).

The richness patterns of bacterial taxa were generally consistent with those observed for relative abundance, with abundant and moderate bacterial taxa increasing across monocropping durations, while rare bacterial taxa gradually declined (Supplementary Figures 4A–C). Similarly, the richness of abundant fungal taxa increased with monocropping duration, whereas no significant differences were detected in the richness of rare fungal taxa among treatments (Supplementary Figures 4D–F).

In addition, LEfSe analysis identified distinct bacterial and fungal biomarkers associated with different monocropping durations (Supplementary Figure 5). For example, M1 was enriched in class Actinobacteria, M3 was associated with genus *Nitrosospira* and phylum Mortierellomycota, whereas M5 was characterized by class Acidobacteriae and order Onygenales.

### 3.4 Microbial co-occurrence network topological properties

**FIGURE 5:**
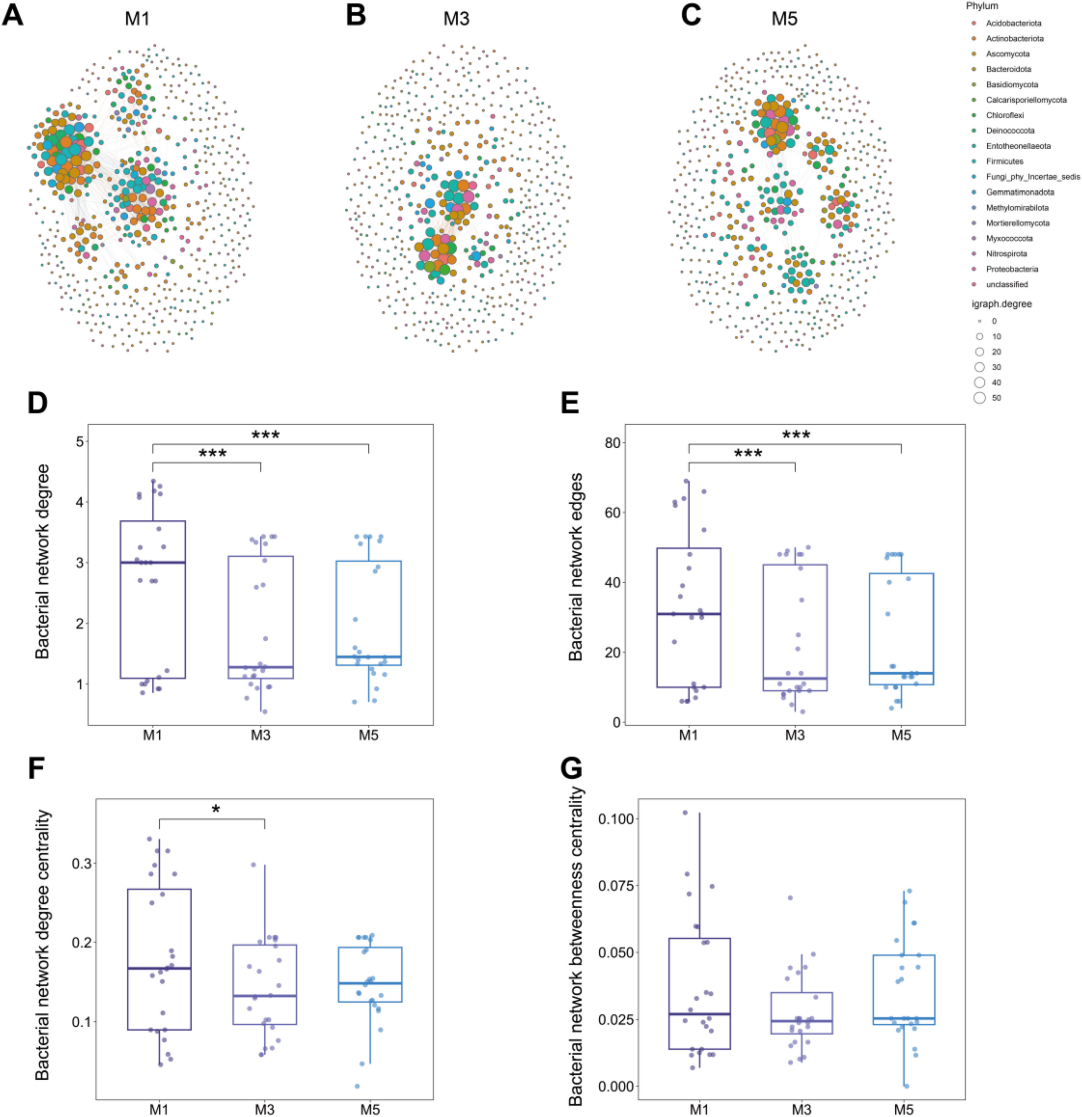
Visualization of microbial co-occurrence network in M1 **(A)**, M3 **(B)**, M5 **(C)** treatments. Boxplots and raw data points of average degree **(D)**, total edges **(E)**, degree centrality **(F)** and betweenness centrality **(G)** of bacterial subnetwork at different monocropping durations of M1 (year 1), M3 (year 3) and M5 (year 5). Boxplots **(D–G)** indicate median (box center line), 25th (box bottom line), 75th (box top line) percentiles, and 5th (the lower whisker) and 95th (the upper whisker) percentiles. Significant differences among treatments are determined based on estimated marginal means (±95% confidence intervals) derived from the linear mixed-effects model followed a *post hoc* Tukey’s HSD test. Significance is indicated by *P < 0.05, ***P < 0.001.

Bacterial–fungal co-occurrence networks were constructed using 481 bacterial ASVs and 109 fungal ASVs, and separate microbial networks were generated for the M1, M3, and M5 groups (Figures 5A–C). In bacterial subnetworks, the average degree, total number of edges, and degree centrality were lower in fields with longer monocropping durations, whereas betweenness centrality showed no clear trend among treatments (Figures 5D–G). By contrast, fungal subnetworks exhibited relatively limited variation across monocropping durations, with only betweenness centrality showing a decreasing trend from M1 to M5 (Supplementary Figure 6). Overall, bacterial subnetworks displayed reduced network complexity in fields with longer monocropping durations, whereas fungal subnetworks appeared comparatively stable.

### 3.5 Soil function in response to different monocropping durations

**FIGURE 6:**
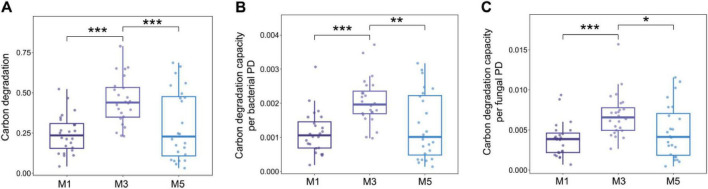
Boxplots and raw data points of carbon degradation **(A)**, carbon degradation capacity per bacterial phylogenetic diversity **(B)** and carbon degradation capacity per fungal phylogenetic diversity **(C)** at different monocropping durations of M1 (year 1), M3 (year 3) and M5 (year 5). Boxplots **(A–C)** indicate median (box center line), 25th (box bottom line), 75th (box top line) percentiles, and 5th (the lower whisker) and 95th (the upper whisker) percentiles. Significant differences among treatments are determined based on estimated marginal means (±95% confidence intervals) derived from the linear mixed-effects model followed a *post hoc* Tukey’s HSD test. “PD” means phylogenetic diversity. Significance is indicated by *P < 0.05, **P < 0.01, ***P < 0.001.

Within each region, the average values of individual soil physical, chemical, and biological parameters and their comparisons among M1, M3, and M5 are presented in Supplementary Table 3. Based on the results of the mixed-effects models, the M3 treatment exhibited the highest carbon degradation function (Figure 6A). No significant differences were observed among treatments for nutrient cycling, physical structure, or carbon storage functions (Supplementary Figures 7A–C). Further analysis showed that carbon degradation capacity normalized by bacterial and fungal phylogenetic diversity was also highest in M3, significantly exceeding the values observed in M1 and M5 (Figures 6B,C). These results indicate a non-linear response of carbon degradation function across monocropping durations, characterized by a transient peak in M3. In addition, the elevated carbon degradation capacity observed in M3 may be associated with a relatively limited subset of bacterial and fungal taxa. By contrast, physical structure, nutrient cycling, and carbon storage functions remained comparatively stable across monocropping durations under long-term bio-organic fertilization.

Site-specific analyses revealed partially distinct functional patterns among locations. In all three sites, carbon degradation function reached its highest value in M3, whereas physical structure and carbon storage showed relatively limited variation among treatments (Supplementary Figure 8). In Tuyugou town, nutrient cycling, physical structure, and carbon storage tended to be lower in fields with longer monocropping durations, whereas carbon degradation peaked in M3 (Supplementary Figure 8). In contrast, carbon degradation and nutrient cycling were higher in fields with longer monocropping durations in Yaerzhen town (Supplementary Figure 8).

Despite these site-specific differences, a general trend toward reduced functional differentiation was observed across monocropping durations. Bray–Curtis dissimilarities and β-dispersion calculated from integrated ecosystem function matrices both declined in fields with longer monocropping durations (Supplementary Figure 9), consistent with reduced functional heterogeneity among treatments. In addition, soil pH and EC both tended to decline across monocropping durations (Supplementary Figure 10).

## 4 Discussion

### 4.1 Putative fungal pathogens are suppressed

*Fusarium*, *Monosporascus* and *Alternaria* are among the most important soil-borne putative fungal pathogens and can substantially affect the growth and productivity of fruit crops and other agricultural plants (Aleandri et al., 2017; Hanson et al., 2018). Consistent with our first hypothesis, fields with longer monocropping durations under long-term bio-organic fertilization showed lower abundances of putative fungal pathogens and higher abundances of beneficial bacterial taxa (Figures 1, 2, Supplementary Figure 1), which is in line with previous studies (Li H. L. et al., 2024; Yang et al., 2025). The pronounced reduction in total putative fungal pathogens, as well as key pathogenic genera such as *Fusarium*, *Monosporascus*, and *Alternaria*, in the M3 and M5 groups is consistent with the possibility that prolonged bio-organic fertilization may contribute to a soil environment less favorable for pathogen proliferation. Previous studies have similarly reported that sustained application of bio-organic fertilizers formulated with *Bacillus subtilis* or *Trichoderma* species can suppress soil-borne diseases in several crop species, including tomato, banana, and watermelon (Pan et al., 2025; Tao et al., 2020; Zhang et al., 2022).

The observed suppression of putative fungal pathogens may be associated with enhanced microbial competition and antagonistic interactions mediated by potentially beneficial bacterial taxa, including *Bacillus*, *Paenibacillus*, *Sphingomonas*, and *Lysobacter* (Cha et al., 2016; Mendes et al., 2011; Wang et al., 2013). However, the mechanisms by which long-term bio-organic fertilization simultaneously suppresses putative fungal pathogens while enriching beneficial bacterial taxa remain unclear and require further investigation. These inferences are based on relative abundance estimates derived from amplicon sequencing, future studies combining sequencing with quantification approaches (e.g., qPCR) are needed to determine whether these shifts correspond to changes in pathogen abundance.

### 4.2 Homogenization of microbial community and simplification of microbial network

Consistent with our second hypothesis, bacterial and fungal communities became increasingly homogenized across fields with longer monocropping durations (Figures 3A,B). In addition, homogeneous selection contributed more strongly to fungal community assembly in M3 than in the other groups (Figure 3F). Recent meta-analysis with large-scale spatial sampling across different land-use types demonstrated that strong homogeneous selection drives bacterial and fungal communities (Peng Z. et al., 2024). However, in the present study, we did not detect a parallel increase in homogeneous selection acting on the bacterial community. Instead, the observed convergence of bacterial assemblages may be associated with the reduced differentiation of multiple ecosystem functions, including carbon storage, physical structure, carbon degradation, and nutrient cycling (Supplementary Figure 9), which paralleled the homogenization patterns observed in bacterial communities. These patterns are consistent with the possibility that long-term fertilization may contribute to reduced environmental differentiation among fields.

An important feature of microbial homogenization was the enrichment of abundant taxa accompanied by a reduction in rare taxa across monocropping durations (Figure 4, Supplementary Figure 4). Rare taxa are often habitat-specific and highly sensitive to environmental change. Despite their relatively low abundance, rare taxa are suggested to disproportionately contribute to ecosystem functioning (Jiao et al., 2016; Ziegler et al., 2018). They may also act as microbial seed banks that help maintain community stability under environmental perturbations. Previous studies have shown that intensive management practices often homogenize microbial communities and reduce rare taxa (Delgado-Baquerizo et al., 2021; Gossner et al., 2016). Given the potential importance of rare taxa and microbial network complexity in supporting ecosystem functioning, these community shifts may have implications for the maintenance of long-term stability of soil functions (Banerjee et al., 2024; Peng Z. et al., 2024).

Microbial co-occurrence network analysis further revealed contrasting responses of bacterial and fungal subnetworks across monocropping durations. Bacterial subnetworks displayed reduced connectivity and centrality in fields with longer monocropping durations, indicating simplified network structures (Figures 5D–F). This pattern coincided with changes in the carbon degradation function, whereas other soil ecosystem functions remained comparatively stable (Figure 6A). Together, these findings suggest that bacterial network structure may respond more sensitively than ecosystem functioning under prolonged monocropping combined with long-term bio-organic fertilization. In addition, previous studies have shown that more complex microbial networks are often associated with higher ecosystem functioning (Zhai et al., 2024), further suggesting that simplified microbial networks may be associated with shifts in soil functional dynamics.

By contrast, fungal subnetworks remained comparatively stable across monocropping durations (Supplementary Figure 6). One possible explanation is that bacteria generally have smaller cell sizes, shorter generation times, and higher substrate uptake rates than fungi, allowing bacterial communities to reorganize interspecific interactions more rapidly under long-term bio-organic fertilization (Fierer et al., 2003). However, fungal responses are inherently delayed due to the time required for hyphal extension and extracellular enzyme production, as well as their stronger dependence on soil pore space and aggregate structure to establish mycelial networks, which imposes greater spatial constraints and consequently slows fungal network reconfiguration (Wang and Kuzyakov, 2024). Another mechanism behind these results might be due to the sampling sites being in a relatively arid area, which was located in northwestern China. Soil bacterial network was less stable and easily disturbed under drought than fungal network (de Vries et al., 2012, 2018). Together, these mechanisms suggest that bacterial networks are more responsive and less stable than fungal networks under prolonged monocropping.

In addition, differences in the biochemical composition of bacterial and fungal necromass may also contribute to the observed patterns in carbon degradation. Bacterial cell walls are primarily composed of relatively labile peptidoglycan, whereas fungal cell walls contain more recalcitrant compounds such as chitin and β-glucans (Torrens and Cava, 2024). Consequently, rapid turnover and decomposition of bacterial-derived organic matter may contribute more strongly to short-term SOC mineralization dynamics than fungal-derived residues.

### 4.3 Changes in carbon degradation but not in other soil functions

Contrary to our third hypothesis, carbon degradation function did not decline consistently across monocropping durations, but instead reached its highest value in M3 (Figure 6A), indicating a non-linear response pattern. Similar intermediate peaks in ecosystem functioning under monocropping systems have also been reported in previous studies (Zheng et al., 2024).

The higher carbon degradation capacity normalized by phylogenetic diversity in M3 suggests that this functional peak may be associated with a relatively limited subset of microbial taxa (Figures 6B, C). Such a pattern may reflect functional redundancy within the microbial community, whereby carbon degradation processes are maintained or even enhanced by a relatively small number of functionally important microorganisms despite reductions in overall community complexity. These findings indicate that changes in microbial community structure and ecosystem functioning may not occur synchronously under prolonged monocropping combined with long-term bio-organic fertilization. However, the specific microbial taxa and metabolic mechanisms responsible for the elevated carbon degradation observed in M3 remain unresolved and require further investigation using approaches that directly assess microbial functional potential and activity.

Overall, these results suggest that monocropping combined with long-term bio-organic fertilization may be associated with microbial community homogenization and simplified bacterial network structures, while most soil ecosystem functions remained comparatively stable across monocropping durations. Because all sampled fields received the same *Bacillus subtilis*–based bio-organic fertilizer, the present field-comparison design cannot fully disentangle the effects of monocropping duration from the cumulative effects of repeated bio-organic fertilization. Therefore, the observed microbial and functional patterns should be interpreted as responses associated with the combined management regime rather than monocropping alone.

## 5 Conclusion

**FIGURE 7:**
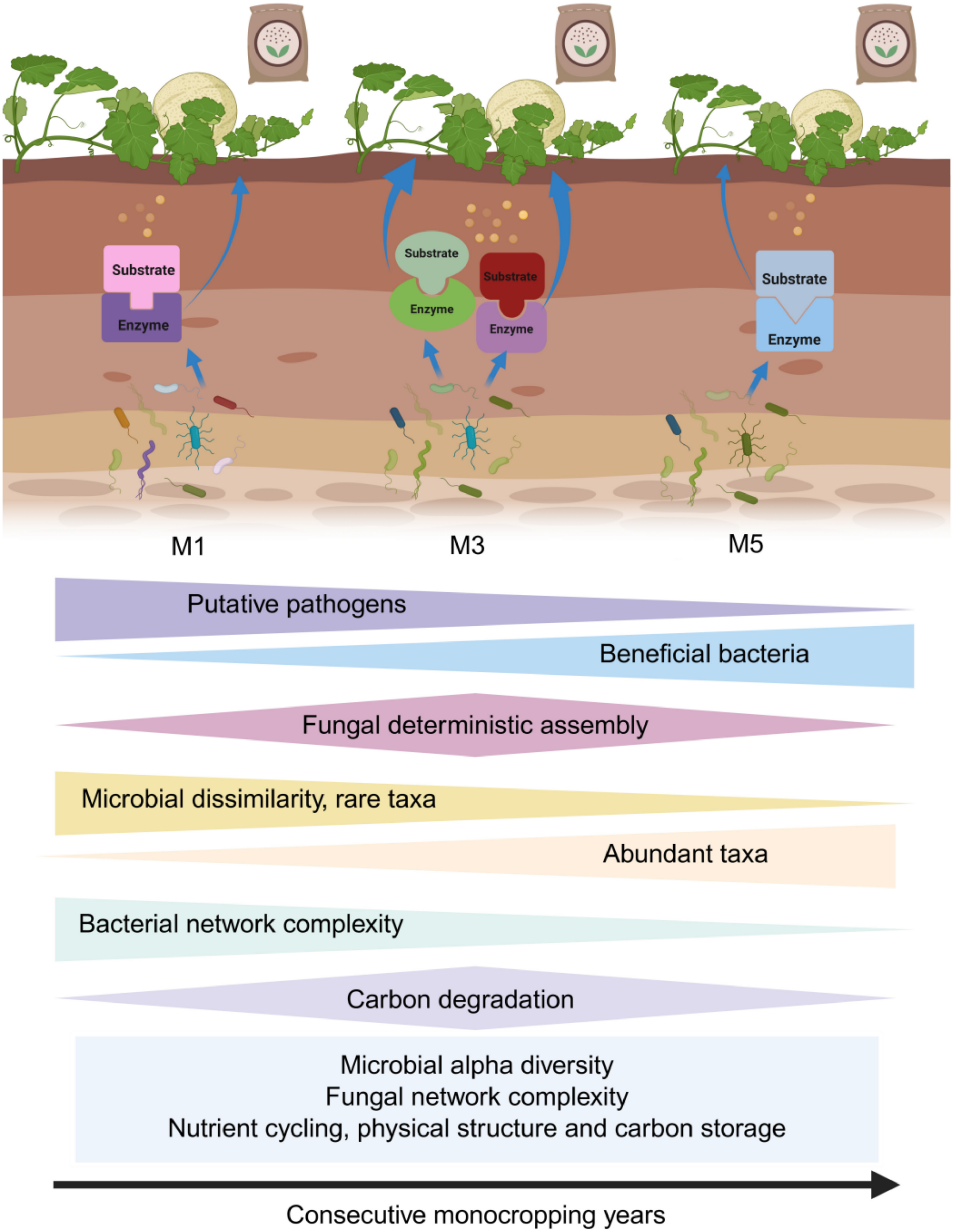
Conceptual model illustrating the effects of consecutive monocropping duration on microbial composition, different microbial taxa, microbial assembly, microbial network complexity and soil ecosystem functions.

In this field-comparison study based on melon fields with different monocropping durations sampled within the same year, long-term application of a *Bacillus subtilis*–based bio-organic fertilizer was associated with reduced abundances of putative fungal pathogens and enrichment of potentially beneficial bacterial taxa. Across fields with longer monocropping durations, microbial communities became increasingly homogenized, accompanied by reduced representation of rare taxa and simplified bacterial co-occurrence network structures, whereas fungal subnetworks remained comparatively stable. In contrast to these pronounced microbial structural shifts, most soil ecosystem functions, including nutrient cycling, carbon storage, and physical structure, remained relatively stable across monocropping durations, although carbon degradation function exhibited a non-linear response pattern with a transient peak in M3. Overall, these microbial and functional responses are summarized in the conceptual model shown in Figure 7. Together, these findings suggest that prolonged monocropping combined with long-term bio-organic fertilization may be associated with stronger responses in microbial community structure than in overall ecosystem functioning.

## Data Availability

The datasets presented in this study can be found in online repositories. The names of the repository/repositories and accession number(s) can be found in the article/Supplementary material. The raw sequencing data generated in this study have been deposited in the NCBI Sequence Read Archive (SRA) under BioProject accession number PRJNA1188318 and BioSample accession number SAMN44831188. All non-sequencing data, including soil enzyme activity datasets, metadata, and processed data files, have been deposited in the Figshare repository (doi: 10.6084/m9.figshare.32614608), which is publicly accessible.
