# Supplementary material for: Consecutive melon monocropping is associated with microbial community homogenization and reduced bacterial network complexity
Source: Front Microbiol. 2026 Jul 27;17:1884506. doi: 10.3389/fmicb.2026.1884506 (PMC13454243; doi:10.3389/fmicb.2026.1884506)
Supplement: Supplementary file 1 [file Table_1.docx]

**Supplementary material**

**Consecutive melon monocropping is associated with microbial community homogenization and reduced bacterial network complexity**

**Supplementary Figures**

Fig. S1 Spearman correlations between the relative abundance of four types of beneficial bacteria and the abundance of total putative pathogens and three pathogen types at the genus level. Significance indicated as **P* < 0.05, ***P* < 0.01, ****P* < 0.001 and “ns” for no significance.

Fig. S2. Distance-decay curves of soil bacterial (A) and fungal community (B) at year 1 (M1), year 3 (M3) and year (M5). Pairwise community similarity is plotted against geographic distance to evaluate how microbial community composition changes with spatial separation. Each curve in plots (A and B) represents one monoculture duration, and the fitted decay slopes indicate the rate at which the community similarity declines with increasing distance. Principal coordinate analysis (PCoA) of bacterial (C) and fungal community structure (D), based on the Bray–Curtis distances. β-dispersion of bacterial (E) and fungal (F) communities under 1-, 3-, and 5-year melon monocropping (M1, M3, and M5, respectively), calculated based on Bray–Curtis distances.

Fig. S3. Boxplots and raw data points of bacterial phylogenetic diversity (A) and fungal phylogenetic diversity (B) at different monoculture years of M1 (year 1), M3 (year 3) and M5 (year 5). Boxplots indicate median (box center line), 25th (box bottom line), 75th (box top line) percentiles, and 5th (the lower whisker) and 95th (the upper whisker) percentiles. Significant differences among treatments were determined based on estimated marginal means (± 95% confidence intervals) derived from the linear mixed-effects model followed a post-hoc Tukey’s HSD test. “ns” means no significance.

Fig. S4. Boxplots and raw data points of abundant bacterial richness (A), moderate bacterial richness (B), rare bacterial richness (C), abundant fungal richness (D), moderate fungal richness (E) and rare fungal richness (F) at different monoculture years of M1 (year 1), M3 (year 3) and M5 (year 5). Boxplots indicate median (box center line), 25th (box bottom line), 75th (box top line) percentiles, and 5th (the lower whisker) and 95th (the upper whisker) percentiles. Significant differences among treatments were determined based on estimated marginal means (± 95% confidence intervals) derived from the linear mixed-effects model followed a post-hoc Tukey’s HSD test. “ns” means no significance. Significance is indicated by **P* < 0.05, ***P* < 0.01, ****P* < 0.001.

Fig. S5. Differentially enriched bacterial (A) and fungal (B) taxa identified by LEfSe analysis across different monoculture durations of M1 (year1), M3 (year 3) and M5 (year 5). Linear discriminant analysis (LDA) is used to determine the taxa that are significantly enriched at each monoculture year. Bars represent taxonomic groups with LDA scores above the significance threshold (log10 scale), indicating their relative contribution to group differentiation. Different colors denote taxa enriched in M1, M3, and M5, respectively. Higher LDA scores indicate stronger discriminatory power of the corresponding taxa between treatments.

Fig. S6. Average degree, total edges, degree centrality and betweenness centrality of fungal subnetwork at different monoculture year of M1 (year1), M3 (year 3) and M5 (year 5). Boxplots and raw data points of average degree (A), total edges (B), degree centrality (C) and betweenness centrality (D) of fungal network at different monoculture durations. Boxplots (A–D) indicate median (box center line), 25th (box bottom line), 75th (box top line) percentiles, and 5th (the lower whisker) and 95th (the upper whisker) percentiles. Significant differences among treatments are determined based on estimated marginal means (± 95% confidence intervals) derived from the linear mixed-effects model followed a post-hoc Tukey’s HSD test. “ns” means no significance. Significance is indicated by **P* < 0.05, ***P* < 0.01, ****P* < 0.001.

Fig. S7. Boxplots and raw data points of nutrient cycling (A), physical structure (B) and carbon storage (C) at different monoculture years of M1 (year 1), M3 (year 3) and M5 (year 5). Boxplots indicate median (box center line), 25th (box bottom line), 75th (box top line) percentiles, and 5th (the lower whisker) and 95th (the upper whisker) percentiles. Significant differences among treatments were determined based on estimated marginal means (± 95% confidence intervals) derived from the linear mixed-effects model followed a post-hoc Tukey’s HSD test. “ns” means no significance.

Fig. S8. Boxplots and raw data points of carbon degradation (A), nutrient cycling (B), physical structure (C) and carbon storage (D) of different monoculture years of M1 (year 1), M3 (year 3) and M5 (year 5) at each sampling site. Boxplots indicate median (box center line), 25th (box bottom line), 75th (box top line) percentiles, and 5th (the lower whisker) and 95th (the upper whisker) percentiles. Significant differences among treatments were determined based on pairwise Kruskal–Wallis tests. “ns” means no significance. Significance is indicated by **P* < 0.05, ***P* < 0.01, ****P* < 0.001.

Fig. S9. Bray–Curtis dissimilarity of all ecosystem functions at different monoculture years of M1 (year 1), M3 (year 3) and M5 (year 5), with statistical differences indicated by different letters (A). β-dispersion of all ecosystem functions of M1 (year 1), M3 (year 3) and M5 (year 5), calculated based on Bray–Curtis distances (B).

Fig. S10. Boxplots and raw data points of soil pH (A) and soil EC (B) at different monoculture years of M1 (year 1), M3 (year 3) and M5 (year 5). Boxplots indicate median (box center line), 25th (box bottom line), 75th (box top line) percentiles, and 5th (the lower whisker) and 95th (the upper whisker) percentiles. Significant differences among treatments were determined based on estimated marginal means (± 95% confidence intervals) derived from the linear mixed-effects model followed a post-hoc Tukey’s HSD test. “ns” means no significance. Significance is indicated by **P* < 0.05, ***P* < 0.01, ****P* < 0.001.

**Supplementary tables**

Table S1 Effects of site and monoculture year on the community structure of bacteria and fungi based on PERMANOVA

| Factors | Bacteria | | Fungi | |
| --- | --- | --- | --- | --- |
|  | *R^2^* | *P* | *R^2^* | *P* |
| Site | 0.149 | 0.001 | 0.197 | 0.001 |
| Year | 0.111 | 0.001 | 0.120 | 0.001 |
| Site: Year | 0.153 | 0.001 | 0.159 | 0.001 |

Table S2 Average relative contributions (%) of five ecological processes to bacterial and fungal community assembly at different monoculture year of M1 (year1), M3 (year 3) and M5 (year 5).

| Group | Bacterial community assembly (%) | | | Fungal community assembly (%) | | |
| --- | --- | --- | --- | --- | --- | --- |
|  | M1 | M3 | M5 | M1 | M3 | M5 |
| HoS | 14.4 | 10.4 | 12.7 | 28.1 | 43.7 | 21.9 |
| HeS | 0.8 | 0 | 2.6 | 1.1 | 0 | 1.2 |
| HD | 4.0 | 1.7 | 1.6 | 0.7 | 0.02 | 0.1 |
| DL | 46.3 | 53.1 | 40.6 | 48.1 | 37.8 | 41.1 |
| DR | 34.6 | 34.8 | 42.4 | 22.0 | 18.5 | 35.6 |

‘DL’: dispersal limitation, ‘DR’: drift, ‘HoS’: homogeneous selection, ‘HD’: homogenizing dispersal and ‘HeS’: heterogeneous selection.

Table S3 Average values and comparison of soil physical, chemical and biological parameters in three regions

| Indicators | Tuyugou | | | Sanbaoxiang | | | Yaerzhen | | |
| --- | --- | --- | --- | --- | --- | --- | --- | --- | --- |
|  | M1 | M3 | M5 | M1 | M3 | M5 | M1 | M3 | M5 |
| Surface hardness | 96.68 ± 37.63 b | 272.76 ± 60.27 a | 279.00 ± 58.90 a | 341.08 ± 51.73 ab | 239.16 ± 62.33 b | 469.06 ± 40.98 a | 289.78 ± 30.57 b | 476.78 ± 55.87 a | 278.79 ± 26.12 b |
| Subsurface hardness | 380.91 ± 115.50 a | 754.64 ± 172.63 a | 679.09 ± 67.96 a | 1289.33 ± 142.54 a | 861.85 ± 171.32 a | 1056.34 ± 102.64 a | 855.76 ± 37.49 a | 661.28 ± 73.89 b | 555.85 ± 48.53 b |
| Bulk density | 1.34 ± 0.03 b | 1.43 ± 0.03 a | 1.45 ± 0.03 a | 1.34 ± 0.03 ab | 1.30 ± 0.04 b | 1.41 ± 0.03 a | 1.40 ± 0.03 a | 1.36 ± 0.04 a | 1.37 ± 0.04 a |
| pH | 8.05 ± 0.06 a | 8.07 ± 0.05 a | 7.97 ± 0.06 a | 8.40 ± 0.09 a | 7.90 ± 0.02 b | 8.02 ± 0.03 b | 7.96 ± 0.03 b | 8.10 ± 0.03 a | 7.83 ± 0.02 c |
| EC | 1.81 ± 0.14 a | 1.00 ± 0.16 b | 1.15 ± 0.20 b | 3.17 ± 0.36 a | 1.17 ± 0.11 b | 1.03 ± 0.07 b | 0.94 ± 0.19 b | 0.76 ± 0.09 b | 1.48 ± 0.13 a |
| TC | 17.74 ± 1.05 a | 15.93 ± 1.16 a | 11.47 ± 1.30 b | 14.69 ± 1.45 a | 13.12 ± 0.87 a | 13.52 ± 0.97 a | 12.94 ± 0.73 a | 12.63 ± 1.62 a | 13.50 ± 1.70 a |
| TN | 8.85 ± 0.49 a | 7.69 ± 0.32 b | 6.27 ± 0.35 c | 4.42 ± 0.30 b | 6.14 ± 0.30 a | 4.29 ± 0.15 b | 4.84 ± 0.23 b | 4.27 ± 0.25 ab | 6.41 ± 0.31 a |
| NH_4_^+^ | 24.64 ± 5.39 a | 16.59 ± 0.99 ab | 12.83 ± 1.93 b | 10.32 ± 0.91 b | 14.69 ± 1.26 a | 10.44 ± 1.14 b | 14.03 ± 0.96 b | 18.13 ± 1.27 ab | 20.28 ± 2.16 a |
| NO_3_^-^ | 85.62 ± 4.10 a | 78.27 ± 3.91 a | 64.11 ± 3.09 b | 43.83 ± 4.12 b | 61.34 ± 3.91 a | 41.74 ± 2.08 b | 45.01 ± 3.66 b | 34.05 ± 2.17 c | 59.27 ± 2.61 a |
| AP | 22.41 ± 3.18 a | 20.59 ± 1.51 a | 18.19 ± 1.60 a | 10.68 ± 1.23 b | 58.76 ± 9.96 a | 17.69 ± 0.59 b | 20.14 ± 1.62 b | 15.89 ± 2.21 b | 39.11 ± 3.76 a |
| AK | 349.25 ± 21.81 a | 266.25 ± 18.84 b | 282.00 ± 21.34 b | 190.38 ± 10.76 b | 273.75 ± 19.92 a | 190.00 ± 9.71 b | 128.13 ± 5.83 c | 193.63 ± 9.84 b | 278.38 ± 28.42 a |
| Exchangeable Ca | 2298.09 ± 92.53 c | 3032.55 ± 44.63 a | 2637.77 ± 146.66 b | 1023.04 ± 124.16 b | 1534.06 ± 115.89 a | 1286.57 ± 63.82 ab | 1829.65 ± 192.53 a | 1814.62 ± 278.53 a | 2148.79 ± 60.64 a |
| Exchangeable Mg | 1129.80 ± 44.03 a | 905.70 ± 29.15 b | 781.50 ± 37.18 c | 1010.40 ± 62.91 a | 836.10 ± 31.87 b | 524.40 ± 12.35 c | 307.20 ± 32.91 a | 412.80 ± 61.34 a | 412.50 ± 54.97 a |
| Cu | 1.11 ± 0.03 a | 1.08 ± 0.04 a | 1.05 ± 0.04 a | 1.21 ± 0.04 a | 0.99 ± 0.04 b | 0.87 ± 0.03 c | 1.69 ± 0.15 b | 1.53 ± 0.12 b | 2.86 ± 0.14 a |
| Fe | 2.70 ± 0.11 a | 2.90 ± 0.20 a | 2.89 ± 0.06 a | 4.77 ± 0.33 a | 4.85 ± 0.16 a | 3.76 ± 0.10 b | 5.10 ± 0.21 c | 6.29 ± 0.25 b | 7.05 ± 0.20 a |
| Zn | 0.82 ± 0.08 b | 1.01 ± 0.05 a | 1.01 ± 0.04 a | 0.29 ± 0.03 c | 0.92 ± 0.14 a | 0.64 ± 0.03 b | 0.84 ± 0.06 b | 0.56 ± 0.04 c | 1.00 ± 0.04 a |
| Mn | 21.23 ± 1.97 a | 11.63 ± 0.41 b | 13.94 ± 0.49 b | 9.29 ± 1.40 ab | 11.88 ± 0.86 a | 8.72 ± 0.30 b | 8.03 ± 0.23 b | 9.17 ± 0.66 ab | 9.59 ± 0.32 a |
| B | 4.43 ± 0.64 a | 1.22 ± 0.05 b | 1.42 ± 0.35 b | 12.37 ± 3.52 a | 2.60 ± 0.99 b | 3.69 ± 0.32 b | 2.94 ± 0.30 b | 4.49 ± 1.09 ab | 5.96 ± 0.50 a |
| Na | 1.89 ± 0.15 a | 0.87 ± 0.05 b | 0.86 ± 0.07 b | 2.79 ± 0.74 a | 0.57 ± 0.06 b | 0.49 ± 0.02 b | 0.39 ± 0.04 b | 0.41 ± 0.05 b | 0.61 ± 0.05 a |
| SOC | 9.15 ± 0.44 a | 7.07 ± 0.27 b | 5.82 ± 0.31 c | 4.37 ± 0.22 b | 5.84 ± 0.21 a | 4.31 ± 0.14 b | 4.71 ± 0.12 b | 4.55 ± 0.28 b | 5.87 ± 0.41 a |
| POXC | 0.30 ± 0.02 a | 0.26 ± 0.01 ab | 0.25 ± 0.01 b | 0.14 ± 0.03 a | 0.20 ± 0.03 a | 0.20 ± 0.01 a | 0.12 ± 0.02 a | 0.14 ± 0.01 a | 0.15 ± 0.01 a |
| CO_2_ | 10.65 ± 2.69 a | 13.20 ± 2.21 a | 10.84 ± 3.85 a | 10.22 ± 1.73 b | 24.25 ± 5.20 a | 6.02 ± 0.59 b | 7.73 ± 0.50 a | 6.66 ± 0.58 a | 7.39 ± 0.61 a |
| Protein | 3.83 ± 0.36 a | 1.79 ± 0.06 b | 1.61 ± 0.16 b | 1.05 ± 0.18 b | 1.74 ± 0.16 a | 1.07 ± 0.15 b | 1.35 ± 0.13 b | 1.11 ± 0.09 b | 1.73 ± 0.11 a |
| AG | 0.90 ± 0.14 b | 1.52 ± 0.12 a | 1.14 ± 0.13 ab | 0.65 ± 0.06 b | 1.11 ± 0.10 a | 0.43 ± 0.04 c | 2.67 ± 0.48 a | 3.31 ± 0.29 a | 3.36 ± 0.23 a |
| BG | 7.02 ± 0.96 b | 10.55 ± 0.66 a | 7.13 ± 0.36 b | 5.21 ± 0.61 b | 8.82 ± 0.95 a | 3.56 ± 0.40 b | 6.25 ± 0.57 b | 10.76 ± 1.37 a | 11.68 ± 0.96 a |
| BX | 1.72 ± 0.25 b | 2.51 ± 0.21 a | 1.74 ± 0.22 b | 1.15 ± 0.12 b | 2.08 ± 0.28 a | 0.78 ± 0.07 b | 1.38 ± 0.19 b | 2.55 ± 0.41 a | 2.82 ± 0.19 a |
| BC | 0.85 ± 0.17 b | 1.31 ± 0.14 a | 0.61 ± 0.09 b | 0.41 ± 0.08 b | 0.80 ± 0.17 a | 0.24 ± 0.03 b | 0.54 ± 0.09 b | 1.24 ± 0.20 a | 1.30 ± 0.14 a |
| NAG | 2.59 ± 0.58 a | 1.27 ± 0.08 b | 1.23 ± 0.13 b | 1.70 ± 0.30 a | 2.19 ± 0.32 a | 0.91 ± 0.12 b | 2.30 ± 0.69 a | 2.48 ± 0.30 a | 2.91 ± 0.31 a |
| LAP | 18.92 ± 1.12 a | 27.12 ± 2.10 a | 23.48 ± 4.01 a | 27.74 ± 4.55 ab | 32.15 ± 3.14 a | 18.71 ± 2.53 b | 55.88 ± 9.48 a | 60.12 ± 6.23 a | 69.57 ± 6.45 a |
| ACP | 44.37 ± 4.41 b | 51.33 ± 5.72 ab | 68.21 ± 9.24 a | 66.37 ± 7.79 a | 66.39 ± 1.78 a | 59.31 ± 10.74 a | 54.63 ± 3.98 b | 74.48 ± 3.49 a | 67.88 ± 1.82 a |
| Sulfatase | 0.29 ± 0.03 a | 0.32 ± 0.04 a | 0.26 ± 0.04 a | 0.13 ± 0.02 c | 0.77 ± 0.09 a | 0.34 ± 0.05 b | 0.75 ± 0.15 a | 1.09 ± 0.07 a | 0.98 ± 0.14 a |

Note: Values are means ± standard error (se). Values in a column followed by different lowercase letters are significantly different at *P* < 0.05.
